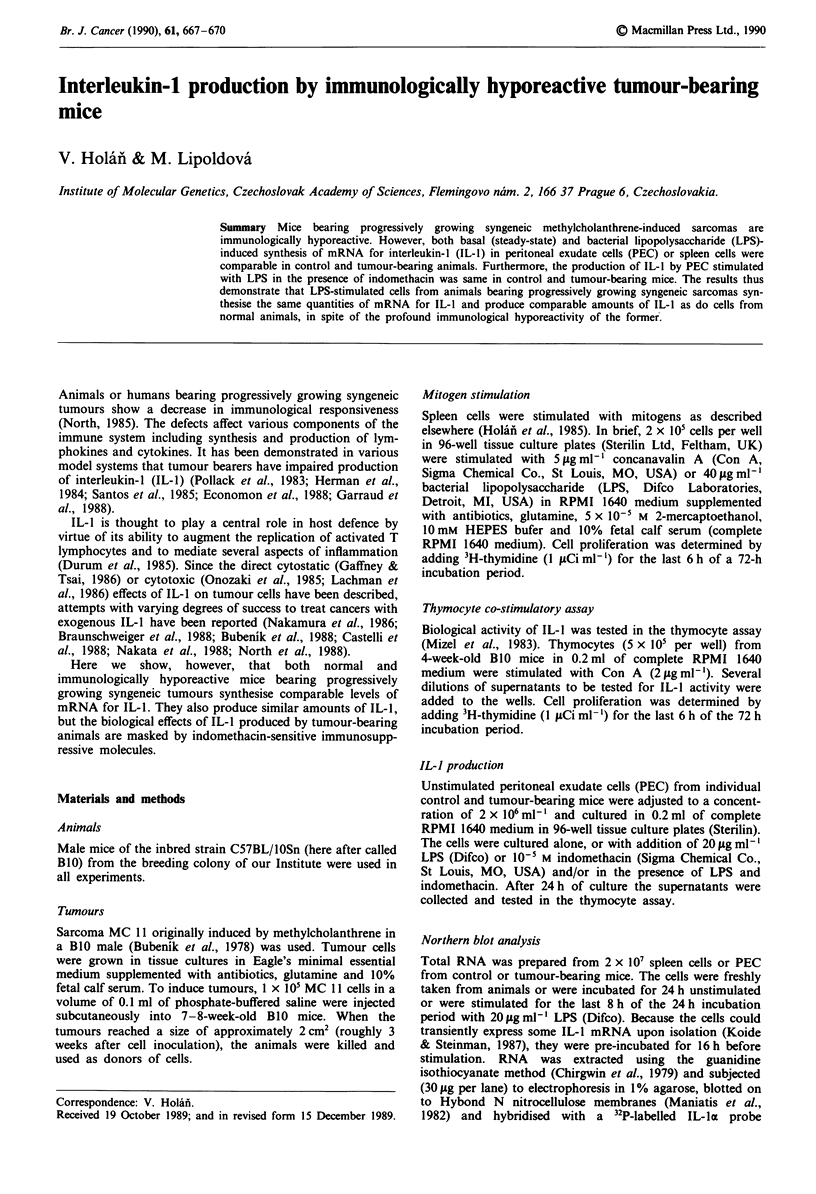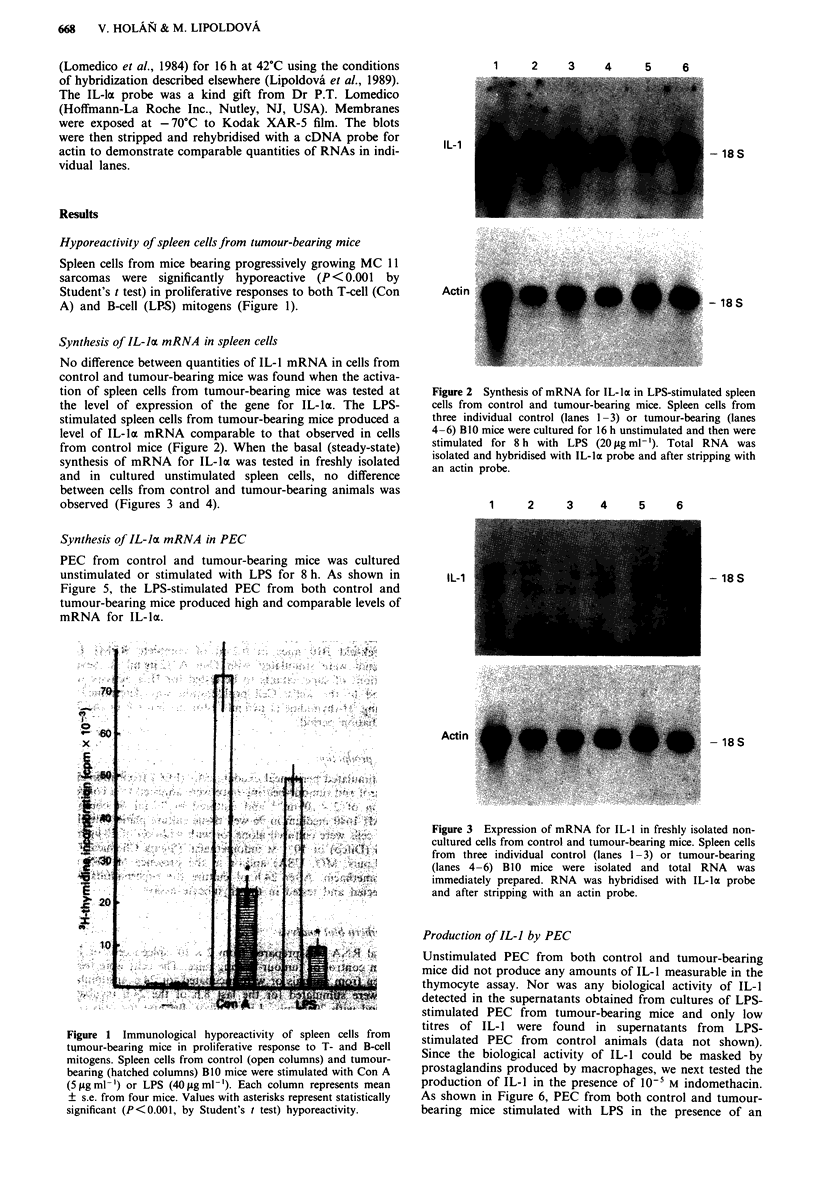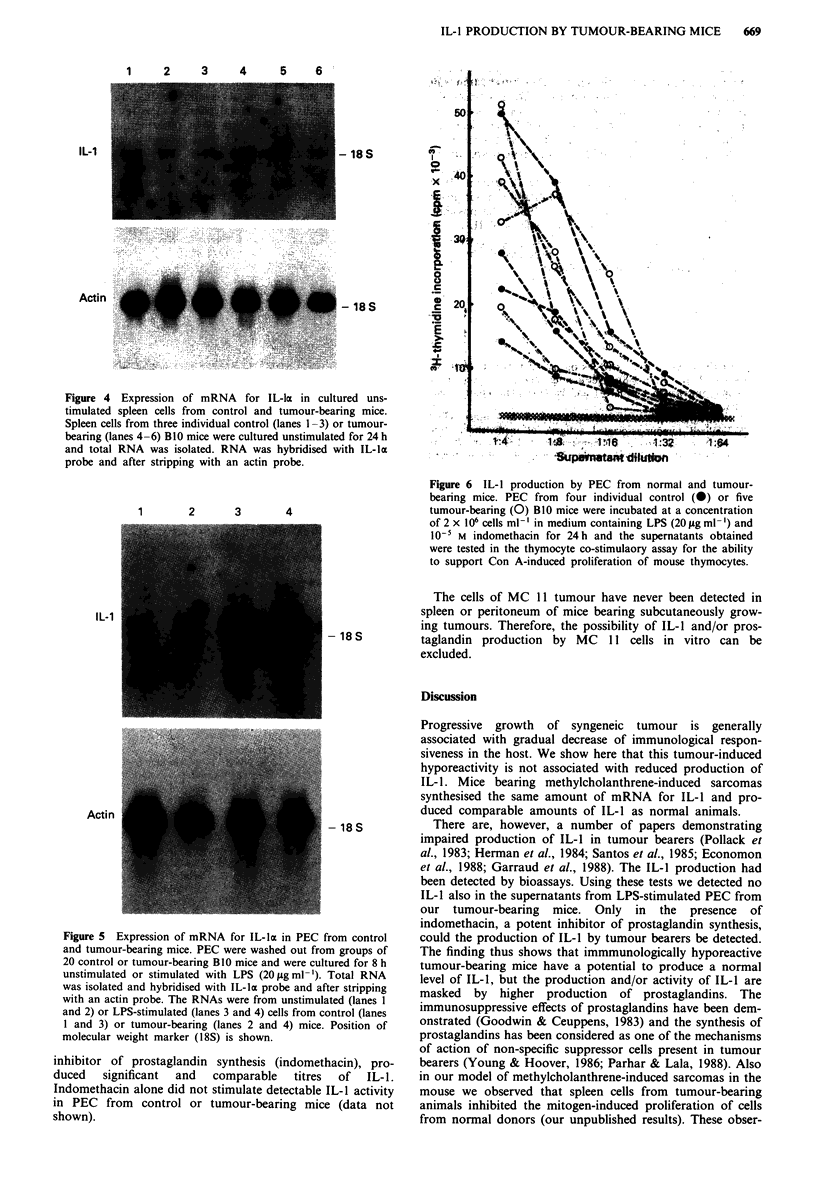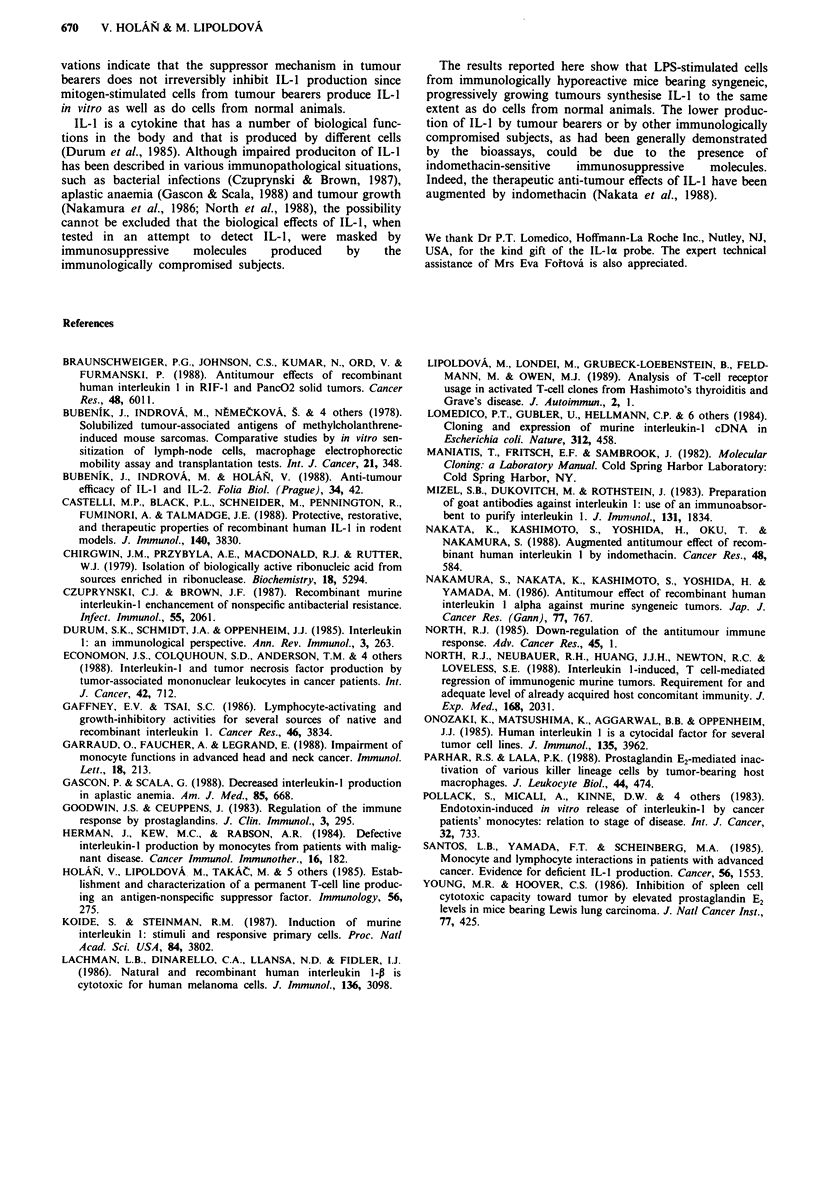# Interleukin-1 production by immunologically hyporeactive tumour-bearing mice.

**DOI:** 10.1038/bjc.1990.151

**Published:** 1990-05

**Authors:** V. Holán, M. Lipoldová

**Affiliations:** Institute of Molecular Genetics, Czechoslovak Academy of Sciences, Prague.

## Abstract

**Images:**


Br. J. Cancer (1990), 61, 667-670 © Macmillan Press Ltd., 1990

### Interleukin-1 production by immunologically hyporeactive tumour-bearing mice

V. Holáň & M. Lipoldová

*Institute of Molecular Genetics, Czechoslovak Academy of Sciences, Flemingovo nám. 2, 166 37 Prague 6, Czechoslovakia.*

**Summary** Mice bearing progressively growing syngeneic methylcholanthrene-induced sarcomas are immunologically hyporeactive. However, both basal (steady-state) and bacterial lipopolysaccharide (LPS)-induced synthesis of mRNA for interleukin-1 (IL-1) in peritoneal exudate cells (PEC) or spleen cells were comparable in control and tumour-bearing animals. Furthermore, the production of IL-1 by PEC stimulated with LPS in the presence of indomethacin was same in control and tumour-bearing mice. The results thus demonstrate that LPS-stimulated cells from animals bearing progressively growing syngeneic sarcomas synthesise the same quantities of mRNA for IL-1 and produce comparable amounts of IL-1 as do cells from normal animals, in spite of the profound immunological hyporeactivity of the former.

Animals or humans bearing progressively growing syngeneic tumours show a decrease in immunological responsiveness (North, 1985). The defects affect various components of the immune system including synthesis and production of lymphokines and cytokines. It has been demonstrated in various model systems that tumour bearers have impaired production of interleukin-1 (IL-1) (Pollack *et al.*, 1983; Herman *et al.*, 1984; Santos *et al.*, 1985; Economon *et al.*, 1988; Garraud *et al.*, 1988).

IL-1 is thought to play a central role in host defence by virtue of its ability to augment the replication of activated T lymphocytes and to mediate several aspects of inflammation (Durum *et al.*, 1985). Since the direct cytostatic (Gaffney & Tsai, 1986) or cytotoxic (Onozaki *et al.*, 1985; Lachman *et al.*, 1986) effects of IL-1 on tumour cells have been described, attempts with varying degrees of success to treat cancers with exogenous IL-1 have been reported (Nakamura *et al.*, 1986; Braunschweiger *et al.*, 1988; Bubeník *et al.*, 1988; Castelli *et al.*, 1988; Nakata *et al.*, 1988; North *et al.*, 1988).

Here we show, however, that both normal and immunologically hyporeactive mice bearing progressively growing syngeneic tumours synthesise comparable levels of mRNA for IL-1. They also produce similar amounts of IL-1, but the biological effects of IL-1 produced by tumour-bearing animals are masked by indomethacin-sensitive immunosuppressive molecules.

### Materials and methods

### *Animals*

Male mice of the inbred strain C57BL/10Sn (here after called B10) from the breeding colony of our Institute were used in all experiments.

### *Tumours*

Sarcoma MC 11 originally induced by methylcholanthrene in a B10 male (Bubeník *et al.*, 1978) was used. Tumour cells were grown in tissue cultures in Eagle's minimal essential medium supplemented with antibiotics, glutamine and 10% fetal calf serum. To induce tumours, $1 \times 10^5$ MC 11 cells in a volume of 0.1 ml of phosphate-buffered saline were injected subcutaneously into 7-8-week-old B10 mice. When the tumours reached a size of approximately 2 $cm^2$ (roughly 3 weeks after cell inoculation), the animals were killed and used as donors of cells.

Correspondence: V. Holáň.
Received 19 October 1989; and in revised form 15 December 1989.

### *Mitogen stimulation*

Spleen cells were stimulated with mitogens as described elsewhere (Holáň *et al.*, 1985). In brief, $2 \times 10^5$ cells per well in 96-well tissue culture plates (Sterilin Ltd, Feltham, UK) were stimulated with 5 $\mu g\ ml^{-1}$ concanavalin A (Con A, Sigma Chemical Co., St Louis, MO, USA) or 40 $\mu g\ ml^{-1}$ bacterial lipopolysaccharide (LPS, Difco Laboratories, Detroit, MI, USA) in RPMI 1640 medium supplemented with antibiotics, glutamine, $5 \times 10^{-5}$ M 2-mercaptoethanol, 10 mM HEPES bufer and 10% fetal calf serum (complete RPMI 1640 medium). Cell proliferation was determined by adding $^3H$-thymidine (1 $\mu Ci\ ml^{-1}$) for the last 6 h of a 72-h incubation period.

### *Thymocyte co-stimulatory assay*

Biological activity of IL-1 was tested in the thymocyte assay (Mizel *et al.*, 1983). Thymocytes ($5 \times 10^5$ per well) from 4-week-old B10 mice in 0.2 ml of complete RPMI 1640 medium were stimulated with Con A (2 $\mu g\ ml^{-1}$). Several dilutions of supernatants to be tested for IL-1 activity were added to the wells. Cell proliferation was determined by adding $^3H$-thymidine (1 $\mu Ci\ ml^{-1}$) for the last 6 h of the 72 h incubation period.

### *IL-1 production*

Unstimulated peritoneal exudate cells (PEC) from individual control and tumour-bearing mice were adjusted to a concentration of $2 \times 10^6\ ml^{-1}$ and cultured in 0.2 ml of complete RPMI 1640 medium in 96-well tissue culture plates (Sterilin). The cells were cultured alone, or with addition of 20 $\mu g\ ml^{-1}$ LPS (Difco) or $10^{-5}$ M indomethacin (Sigma Chemical Co., St Louis, MO, USA) and/or in the presence of LPS and indomethacin. After 24 h of culture the supernatants were collected and tested in the thymocyte assay.

### *Northern blot analysis*

Total RNA was prepared from $2 \times 10^7$ spleen cells or PEC from control or tumour-bearing mice. The cells were freshly taken from animals or were incubated for 24 h unstimulated or were stimulated for the last 8 h of the 24 h incubation period with 20 $\mu g\ ml^{-1}$ LPS (Difco). Because the cells could transiently express some IL-1 mRNA upon isolation (Koide & Steinman, 1987), they were pre-incubated for 16 h before stimulation. RNA was extracted using the guanidine isothiocyanate method (Chirgwin *et al.*, 1979) and subjected (30 $\mu g$ per lane) to electrophoresis in 1% agarose, blotted on to Hybond N nitrocellulose membranes (Maniatis *et al.*, 1982) and hybridised with a $^{32}P$-labelled IL-1$\alpha$ probe

(Lomedico *et al.*, 1984) for 16 h at 42°C using the conditions of hybridization described elsewhere (Lipoldová *et al.*, 1989). The IL-1α probe was a kind gift from Dr P.T. Lomedico (Hoffmann-La Roche Inc., Nutley, NJ, USA). Membranes were exposed at −70°C to Kodak XAR-5 film. The blots were then stripped and rehybridised with a cDNA probe for actin to demonstrate comparable quantities of RNAs in individual lanes.

### Results

### *Hyporeactivity of spleen cells from tumour-bearing mice*

Spleen cells from mice bearing progressively growing MC 11 sarcomas were significantly hyporeactive ($P<0.001$ by Student's *t* test) in proliferative responses to both T-cell (Con A) and B-cell (LPS) mitogens (Figure 1).

### *Synthesis of IL-1α mRNA in spleen cells*

No difference between quantities of IL-1 mRNA in cells from control and tumour-bearing mice was found when the activation of spleen cells from tumour-bearing mice was tested at the level of expression of the gene for IL-1α. The LPS-stimulated spleen cells from tumour-bearing mice produced a level of IL-1α mRNA comparable to that observed in cells from control mice (Figure 2). When the basal (steady-state) synthesis of mRNA for IL-1α was tested in freshly isolated and in cultured unstimulated spleen cells, no difference between cells from control and tumour-bearing animals was observed (Figures 3 and 4).

### *Synthesis of IL-1α mRNA in PEC*

PEC from control and tumour-bearing mice was cultured unstimulated or stimulated with LPS for 8 h. As shown in Figure 5, the LPS-stimulated PEC from both control and tumour-bearing mice produced high and comparable levels of mRNA for IL-1α.

**Figure 1** Immunological hyporeactivity of spleen cells from tumour-bearing mice in proliferative response to T- and B-cell mitogens. Spleen cells from control (open columns) and tumour-bearing (hatched columns) B10 mice were stimulated with Con A (5 μg ml⁻¹) or LPS (40 μg ml⁻¹). Each column represents mean ± s.e. from four mice. Values with asterisks represent statistically significant ($P<0.001$, by Student's *t* test) hyporeactivity.

**Figure 2** Synthesis of mRNA for IL-1α in LPS-stimulated spleen cells from control and tumour-bearing mice. Spleen cells from three individual control (lanes 1–3) or tumour-bearing (lanes 4–6) B10 mice were cultured for 16 h unstimulated and then were stimulated for 8 h with LPS (20 μg ml⁻¹). Total RNA was isolated and hybridised with IL-1α probe and after stripping with an actin probe.

**Figure 3** Expression of mRNA for IL-1 in freshly isolated non-cultured cells from control and tumour-bearing mice. Spleen cells from three individual control (lanes 1–3) or tumour-bearing (lanes 4–6) B10 mice were isolated and total RNA was immediately prepared. RNA was hybridised with IL-1α probe and after stripping with an actin probe.

### *Production of IL-1 by PEC*

Unstimulated PEC from both control and tumour-bearing mice did not produce any amounts of IL-1 measurable in the thymocyte assay. Nor was any biological activity of IL-1 detected in the supernatants obtained from cultures of LPS-stimulated PEC from tumour-bearing mice and only low titres of IL-1 were found in supernatants from LPS-stimulated PEC from control animals (data not shown). Since the biological activity of IL-1 could be masked by prostaglandins produced by macrophages, we next tested the production of IL-1 in the presence of $10^{-5}$ M indomethacin. As shown in Figure 6, PEC from both control and tumour-bearing mice stimulated with LPS in the presence of an

**Figure 4** Expression of mRNA for IL-1α in cultured unstimulated spleen cells from control and tumour-bearing mice. Spleen cells from three individual control (lanes 1–3) or tumour-bearing (lanes 4–6) B10 mice were cultured unstimulated for 24 h and total RNA was isolated. RNA was hybridised with IL-1α probe and after stripping with an actin probe.

**Figure 5** Expression of mRNA for IL-1α in PEC from control and tumour-bearing mice. PEC were washed out from groups of 20 control or tumour-bearing B10 mice and were cultured for 8 h unstimulated or stimulated with LPS (20 μg ml$^{-1}$). Total RNA was isolated and hybridised with IL-1α probe and after stripping with an actin probe. The RNAs were from unstimulated (lanes 1 and 2) or LPS-stimulated (lanes 3 and 4) cells from control (lanes 1 and 3) or tumour-bearing (lanes 2 and 4) mice. Position of molecular weight marker (18S) is shown.

inhibitor of prostaglandin synthesis (indomethacin), produced significant and comparable titres of IL-1. Indomethacin alone did not stimulate detectable IL-1 activity in PEC from control or tumour-bearing mice (data not shown).

**Figure 6** IL-1 production by PEC from normal and tumour-bearing mice. PEC from four individual control (●) or five tumour-bearing (○) B10 mice were incubated at a concentration of 2 × 10$^6$ cells ml$^{-1}$ in medium containing LPS (20 μg ml$^{-1}$) and 10$^{-5}$ M indomethacin for 24 h and the supernatants obtained were tested in the thymocyte co-stimulaory assay for the ability to support Con A-induced proliferation of mouse thymocytes.

The cells of MC 11 tumour have never been detected in spleen or peritoneum of mice bearing subcutaneously growing tumours. Therefore, the possibility of IL-1 and/or prostaglandin production by MC 11 cells in vitro can be excluded.

### Discussion

Progressive growth of syngeneic tumour is generally associated with gradual decrease of immunological responsiveness in the host. We show here that this tumour-induced hyporeactivity is not associated with reduced production of IL-1. Mice bearing methylcholanthrene-induced sarcomas synthesised the same amount of mRNA for IL-1 and produced comparable amounts of IL-1 as normal animals.

There are, however, a number of papers demonstrating impaired production of IL-1 in tumour bearers (Pollack *et al.*, 1983; Herman *et al.*, 1984; Santos *et al.*, 1985; Economon *et al.*, 1988; Garraud *et al.*, 1988). The IL-1 production had been detected by bioassays. Using these tests we detected no IL-1 also in the supernatants from LPS-stimulated PEC from our tumour-bearing mice. Only in the presence of indomethacin, a potent inhibitor of prostaglandin synthesis, could the production of IL-1 by tumour bearers be detected. The finding thus shows that immmunologically hyporeactive tumour-bearing mice have a potential to produce a normal level of IL-1, but the production and/or activity of IL-1 are masked by higher production of prostaglandins. The immunosuppressive effects of prostaglandins have been demonstrated (Goodwin & Ceuppens, 1983) and the synthesis of prostaglandins has been considered as one of the mechanisms of action of non-specific suppressor cells present in tumour bearers (Young & Hoover, 1986; Parhar & Lala, 1988). Also in our model of methylcholanthrene-induced sarcomas in the mouse we observed that spleen cells from tumour-bearing animals inhibited the mitogen-induced proliferation of cells from normal donors (our unpublished results). These observations indicate that the suppressor mechanism in tumour bearers does not irreversibly inhibit IL-1 production since mitogen-stimulated cells from tumour bearers produce IL-1 *in vitro* as well as do cells from normal animals.

IL-1 is a cytokine that has a number of biological functions in the body and that is produced by different cells (Durum *et al.*, 1985). Although impaired produciton of IL-1 has been described in various immunopathological situations, such as bacterial infections (Czuprynski & Brown, 1987), aplastic anaemia (Gascon & Scala, 1988) and tumour growth (Nakamura *et al.*, 1986; North *et al.*, 1988), the possibility cannot be excluded that the biological effects of IL-1, when tested in an attempt to detect IL-1, were masked by immunosuppressive molecules produced by the immunologically compromised subjects.

The results reported here show that LPS-stimulated cells from immunologically hyporeactive mice bearing syngeneic, progressively growing tumours synthesise IL-1 to the same extent as do cells from normal animals. The lower production of IL-1 by tumour bearers or by other immunologically compromised subjects, as had been generally demonstrated by the bioassays, could be due to the presence of indomethacin-sensitive immunosuppressive molecules. Indeed, the therapeutic anti-tumour effects of IL-1 have been augmented by indomethacin (Nakata *et al.*, 1988).

We thank Dr P.T. Lomedico, Hoffmann-La Roche Inc., Nutley, NJ, USA, for the kind gift of the IL-1α probe. The expert technical assistance of Mrs Eva Fořtová is also appreciated.

### References

BRAUNSCHWEIGER, P.G., JOHNSON, C.S., KUMAR, N., ORD, V. & FURMANSKI, P. (1988). Antitumour effects of recombinant human interleukin 1 in RIF-1 and PancO2 solid tumors. *Cancer Res.*, **48**, 6011.

BUBENÍK, J., INDROVÁ, M., NĚMEČKOVÁ, Š. & 4 others (1978). Solubilized tumour-associated antigens of methylcholanthrene-induced mouse sarcomas. Comparative studies by *in vitro* sensitization of lymph-node cells, macrophage electrophorectic mobility assay and transplantation tests. *Int. J. Cancer*, **21**, 348.

BUBENÍK, J., INDROVÁ, M. & HOLÁŇ, V. (1988). Anti-tumour efficacy of IL-1 and IL-2. *Folia Biol. (Prague)*, **34**, 42.

CASTELLI, M.P., BLACK, P.L., SCHNEIDER, M., PENNINGTON, R., FUMINORI, A. & TALMADGE, J.E. (1988). Protective, restorative, and therapeutic properties of recombinant human IL-1 in rodent models. *J. Immunol.*, **140**, 3830.

CHIRGWIN, J.M., PRZYBYLA, A.E., MACDONALD, R.J. & RUTTER, W.J. (1979). Isolation of biologically active ribonucleic acid from sources enriched in ribonuclease. *Biochemistry*, **18**, 5294.

CZUPRYNSKI, C.J. & BROWN, J.F. (1987). Recombinant murine interleukin-1 enchancement of nonspecific antibacterial resistance. *Infect. Immunol.*, **55**, 2061.

DURUM, S.K., SCHMIDT, J.A. & OPPENHEIM, J.J. (1985). Interleukin 1: an immunological perspective. *Ann. Rev. Immunol.*, **3**, 263.

ECONOMON, J.S., COLQUHOUN, S.D., ANDERSON, T.M. & 4 others (1988). Interleukin-1 and tumor necrosis factor production by tumor-associated mononuclear leukocytes in cancer patients. *Int. J. Cancer*, **42**, 712.

GAFFNEY, E.V. & TSAI, S.C. (1986). Lymphocyte-activating and growth-inhibitory activities for several sources of native and recombinant interleukin 1. *Cancer Res.*, **46**, 3834.

GARRAUD, O., FAUCHER, A. & LEGRAND, E. (1988). Impairment of monocyte functions in advanced head and neck cancer. *Immunol. Lett.*, **18**, 213.

GASCON, P. & SCALA, G. (1988). Decreased interleukin-1 production in aplastic anemia. *Am. J. Med.*, **85**, 668.

GOODWIN, J.S. & CEUPPENS, J. (1983). Regulation of the immune response by prostaglandins. *J. Clin. Immunol.*, **3**, 295.

HERMAN, J., KEW, M.C., & RABSON, A.R. (1984). Defective interleukin-1 production by monocytes from patients with malignant disease. *Cancer Immunol. Immunother.*, **16**, 182.

HOLÁŇ, V., LIPOLDOVÁ, M., TAKÁČ, M. & 5 others (1985). Establishment and characterization of a permanent T-cell line producing an antigen-nonspecific suppressor factor. *Immunology*, **56**, 275.

KOIDE, S. & STEINMAN, R.M. (1987). Induction of murine interleukin 1: stimuli and responsive primary cells. *Proc. Natl Acad. Sci. USA*, **84**, 3802.

LACHMAN, L.B., DINARELLO, C.A., LLANSA, N.D. & FIDLER, I.J. (1986). Natural and recombinant human interleukin 1-β is cytotoxic for human melanoma cells. *J. Immunol.*, **136**, 3098.

LIPOLDOVÁ, M., LONDEI, M., GRUBECK-LOEBENSTEIN, B., FELDMANN, M. & OWEN, M.J. (1989). Analysis of T-cell receptor usage in activated T-cell clones from Hashimoto's thyroiditis and Grave's disease. *J. Autoimmun.*, **2**, 1.

LOMEDICO, P.T., GUBLER, U., HELLMANN, C.P. & 6 others (1984). Cloning and expression of murine interleukin-1 cDNA in *Escherichia coli*. *Nature*, **312**, 458.

MANIATIS, T., FRITSCH, E.F. & SAMBROOK, J. (1982). *Molecular Cloning: a Laboratory Manual*. Cold Spring Harbor Laboratory: Cold Spring Harbor, NY.

MIZEL, S.B., DUKOVITCH, M. & ROTHSTEIN, J. (1983). Preparation of goat antibodies against interleukin 1: use of an immunoabsorbent to purify interleukin 1. *J. Immunol.*, **131**, 1834.

NAKATA, K., KASHIMOTO, S., YOSHIDA, H., OKU, T. & NAKAMURA, S. (1988). Augmented antitumour effect of recombinant human interleukin 1 by indomethacin. *Cancer Res.*, **48**, 584.

NAKAMURA, S., NAKATA, K., KASHIMOTO, S., YOSHIDA, H. & YAMADA, M. (1986). Antitumour effect of recombinant human interleukin 1 alpha against murine syngeneic tumors. *Jap. J. Cancer Res. (Gann)*, **77**, 767.

NORTH, R.J. (1985). Down-regulation of the antitumour immune response. *Adv. Cancer Res.*, **45**, 1.

NORTH, R.J., NEUBAUER, R.H., HUANG, J.J.H., NEWTON, R.C. & LOVELESS, S.E. (1988). Interleukin 1-induced, T cell-mediated regression of immunogenic murine tumors. Requirement for and adequate level of already acquired host concomitant immunity. *J. Exp. Med.*, **168**, 2031.

ONOZAKI, K., MATSUSHIMA, K., AGGARWAL, B.B. & OPPENHEIM, J.J. (1985). Human interleukin 1 is a cytocidal factor for several tumor cell lines. *J. Immunol.*, **135**, 3962.

PARHAR, R.S. & LALA, P.K. (1988). Prostaglandin $E_2$-mediated inactivation of various killer lineage cells by tumor-bearing host macrophages. *J. Leukocyte Biol.*, **44**, 474.

POLLACK, S., MICALI, A., KINNE, D.W. & 4 others (1983). Endotoxin-induced *in vitro* release of interleukin-1 by cancer patients' monocytes: relation to stage of disease. *Int. J. Cancer*, **32**, 733.

SANTOS, L.B., YAMADA, F.T. & SCHEINBERG, M.A. (1985). Monocyte and lymphocyte interactions in patients with advanced cancer. Evidence for deficient IL-1 production. *Cancer*, **56**, 1553.

YOUNG, M.R. & HOOVER, C.S. (1986). Inhibition of spleen cell cytotoxic capacity toward tumor by elevated prostaglandin $E_2$ levels in mice bearing Lewis lung carcinoma. *J. Natl Cancer Inst.*, **77**, 425.